# Cystatin SN neutralizes the inhibitory effect of cystatin C on cathepsin B activity

**DOI:** 10.1038/cddis.2013.485

**Published:** 2013-12-19

**Authors:** J-T Kim, S-J Lee, M A Kang, J E Park, B-Y Kim, D-Y Yoon, Y Yang, C-H Lee, Y I Yeom, Y-K Choe, H G Lee

**Affiliations:** 1 Biomedical Genomics Research Center, Korea Research Institute of Bioscience and Biotechnology, Daejeon, Republic of Korea; 2 Chemical Biology Research Center, Korea Research Institute of Bioscience and Biotechnology, Daejeon, Republic of Korea; 3 Department of Bioscience and Biotechnology, Bio/Molecular Informatics Center, Konkuk University, Seoul, Republic of Korea; 4 Department of Life Science, Sookmyung Women's University, Seoul, Republic of Korea; 5 Laboratory Animal Center, Korea Research Institute of Bioscience and Biotechnology, Daejeon, Republic of Korea

**Keywords:** cystatin SN, Cystatin C, Cathepsin B, colon cancer, invasion

## Abstract

Cystatin SN (CST1) is one of the several salivary cystatins that form tight equimolar complexes with cysteine proteases, such as the cathepsins. High expression of CST1 is correlated with advanced pTNM stage in gastric cancer. However, the functional role of CST1 in tumorigenesis has not been elucidated. In this study, we showed that CST1 was highly expressed in colon tumor tissues, compared with nontumor regions. Increased cell proliferation and invasiveness were observed in HCT116 cell lines stably transfected with *CST1* cDNA (HCT116-CST1) but not in *CST3*-transfected cells. We also demonstrated that CST1-overexpressing cell lines exhibited increased tumor growth as well as metastasis in a xenograft nude mouse model. Interestingly, CST1 interacted with cystatin C (CST3), a potent cathepsin B (CTSB) inhibitor, with a higher affinity than the interaction between CST3 and CTSB in the extracellular space of HCT116 cells. CTSB-mediated cellular invasiveness and proteolytic activities were strongly inhibited by CST3, but in the presence of CST1 CTSB activities recovered significantly. Furthermore, domain mapping of CST1 showed that the disulfide-bonded conformation, or conserved folding, of CST1 is important for its secretion and for the neutralization of CST3 activity. These results suggest that CST1 upregulation might be involved in colorectal tumorigenesis and acts by neutralizing the inhibition of CTSB proteolytic activity by CST3.

A number of clinical reports have shown a significant association between enhanced protease activity and poor prognosis,^1, 2^ indicating that proteolytic enzymes that are upregulated in cancer cells may be useful therapeutic targets. Among these proteolytic enzymes, cysteine proteases, such as cathepsins, papain, and calpains, are widely distributed in tissues and have various functions, including degradation of extracellular matrix, modulation of immune response, tissue development, and induction of monocytes and cancer cells.^3, 4, 5^ Cathepsins are intracellular cysteine proteases that function in protein degradation in lysosomes^6^ and secretory granules. In various malignant tumors, cathepsins are overexpressed and localize to the invasive tumor margin.^7^ Because increased expression of cathepsin proteases is significantly correlated with more aggressive tumors and poorer prognosis,^1^ inhibition of cathepsin activity may reduce tumor invasion and growth.^8, 9, 10^

The proteolytic activities of cysteine proteases are controlled by specific inhibitors belonging to the cystatin superfamily.^11^ Cystatins are essential to organisms, because they protect tissues from inappropriate proteolysis. Cystatins compete with protease substrates to form tight equimolar complexes with cysteine proteases by binding to their active sites.^12^ Type-2 cystatin genes (*CST1-5*, *CSTP1* and *CSTP2*), physically clustered at human chromosome 20p11.2,^13, 14^ are phylogenetically diverse, and widely distributed throughout nature,^15, 16^ and usually include a secretory leader peptide. The *CST1*, *CST2*, and *CST4* genes encode the S-type (salivary) cystatins SN, SA, and S, respectively.^16^ The S-type cystatins have been detected by immunoassay in seminal plasma, tears, and tracheobronchial fluid but not in other bodily fluids or secretions.^16, 17^ Although they have protective roles in antibacterial and antiviral processes, the functions of cystatins in these fluids are still unclear. CST1 (cystatin SN) is homologous with the chicken cystatins, CST4 and CST2, having 39–42% identity and 68% functionally conserved residues.^18^ Papain, ficin, cathepsin C (CTSC), and HSV-1 are inhibited by CST1,^15^ but CTSB, a major lysosomal cysteine protease, is not.^19^ Cathepsins B, H, L, and V are inhibited by CST3.^20, 21^ CST3 (cystatin C), a potent inhibitor of CTSB, has a broader spectrum of inhibitory activity than CST1.^22^

CST3 is associated with tumor metastasis and invasion,^23, 24^ and its expression is correlated with a high risk of death in patients with colorectal cancer (CRC).^25^ CST6 is more highly expressed in metastatic cancers than in primary cancers,^26, 27^ and CST7 is upregulated in murine liver metastatic cancers. These data indicate that the role of cystatins as inhibitors of cysteine proteases might be important in regulating the invasion and metastasis of cancer cells. Previously, we reported that *CST1* was upregulated in gastric cancer tissues, compared with nontumor regions, and clinicopathological analysis showed a significant correlation between high expression of CST1 and pathological tumor, node, metastasis stage.^28^

Here, we found that CST1 was highly upregulated in CRC tissues compared with normal tissue regions, and the interaction between CST1 and CST3 was stronger than the binding between CST3 and CTSB in the extracellular space. Finally, we found that the heterodimeric binding between CST1 and CST3 decreases the proteolytic activity of CTSB and cellular invasiveness.

## Results

### Upregulation and R91R-silent mutation of CST1 in CRC tissues and cell lines

**Figure 1:**
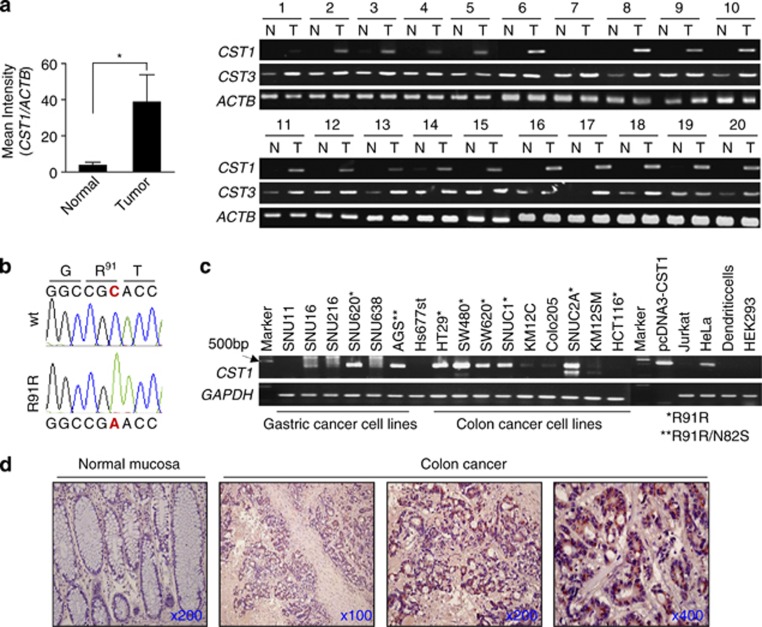
Upregulation and silent mutation (Arg-91) of *CST1* in colon cancer. (**a**) Tissues from 20 patients with CRC were prepared, and RT-PCR analysis was performed. *CST1* was strongly upregulated in tumor (T) tissues compared with nontumor (N) regions. The histogram (left) showing mean±S.D. values indicates the relative band intensity in arbitrary densitometric units. *P<0.05. (**b**) Automatic DNA sequencing of *CST1* cDNA. The CGC (Arg-91) codon was mutated to CGA (Arg) in HT29 cells. (**c**) RT-PCR analysis of *CST1* in colon and gastric cancer cell lines. The pcDNA3.1-CST1 plasmid was used as a positive control for the *CST1* cDNA band (423 bp). *CST1-R91R* mutated cells are indicated by an asterisk. *ACTB* and *GAPDH* were used as reaction controls. (**d**) Representative CST1 protein staining in CRC tissues. Cancerous tissues and normal mucosa were examined by immunohistochemistry using an anti-CST1 antibody

Using a GeneChip microarray, we found that *CST1* was significantly upregulated in CRC tissues, as previously reported in gastric cancer.^28^ The mRNA transcript of *CST1* was highly elevated in CRC tissues (*n*=66, *P*<0.01) compared with nontumor regions (*n*=9), but there were no significant differences in *CST1* expression between recurrent and nonrecurrent tumors or between stage I and II tumors (data not shown). To verify whether *CST1* was significantly upregulated in CRC tissues, we analyzed CRC tissues from 20 patients using RT-PCR (Figure 1a). Consistent with the microarray results, the mRNA of *CST1* was increased approximately 20-fold in cancerous regions (*P*<0.05) but not in normal tissues taken from a distant site. *CST3*, a ubiquitously expressed type-2 cystatin, was only slightly upregulated in cancerous regions. Interestingly, examination of the cDNA sequence of *CST1* showed that the CGC codon encoding Arg-91 (R) was mutated to CGA (silent mutation R91R) in colon cancer tissues (Figure 1b). *CST3* cDNA was not mutated in the coding region. As shown in Figure 1c, *CST1* transcripts were differentially expressed, and many cells, including gastric and CRC cell lines, had the R91R mutation. In AGS cells, *CST1* cDNA was mutated at both R91R and N82S (AAT to AGT). Interestingly, *CST1* expression was not observed in Jurkat (T-cell lymphoma) cells, monocyte-derived dendritic cells, or Hs677.st cells (normal fetal gastric cells). Immunohistochemistry showed upregulation of CST1 protein in tumor tissues from patients with CRC (Figure 1d). Normal colonic mucosa was not stained, but cancerous regions were stained with anti-CST1 antibody, showing accumulation of CST1 in the endoplasmic reticulum and Golgi apparatus.

### CST1 enhances tumor cell growth in xenograft nude mice model

**Figure 2:**
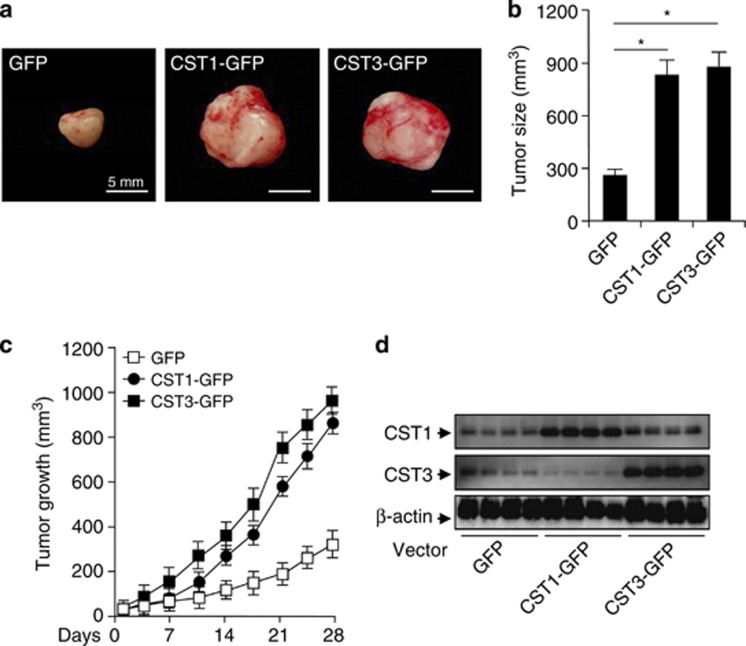
CST1 overexpression increases tumor growth activity in colon cancer xenograft model. HCT-116 cells were injected s.c. into athymic nude mice, which subsequently overexpressed GFP, CST1-GFP, or CST3-GFP for 4 weeks. (**a**) Macroscopic appearance and size of HCT-116 tumors from BALB/c nude mice 4 weeks after implantation of cells. Bar=5 mm. (**b**) The graph shows mean tumor size±S.D. (*P<0.05, Student's *t*-test), *n*=7 mice per treatment group. (**c**) Tumor volume was measured twice a week. Data represent means±S.E.M. from three independent experiments. (*P<0.05, Student's *t*-test). (**d**) Western blotting analysis of total CST1 and CST3 in tumor tissues. The immunoblot shown is representative of three independent experiments. *β*-actin served as the standard

To examine the underlying mechanism(s) by which CST1 increases tumor growth, we monitored the primary growth of CST1- and CST3-overexpressing colon cancer cells using a xenograft assay. HCT-116 cells expressing CST1-GFP or CST3-GFP formed larger tumors in athymic nude mice compared with the control GFP (Figures 2a and b). However, tumor size in CST1-GFP-implanted mice did not show a significant difference with CST3-GFP. In athymic nude mice, we measured tumor growth over time. Implanted CST1-GFP and CST3-GFP cells (4 weeks) each enhanced tumor growth relative to the control (Figure 2c), and we confirmed the higher CST1 and CST3 expression in implanted tumor tissues (Figure 2d). These results showed that CST1 and CST3 expression in CRC contribute to tumor growth.

### Cellular localization and secretion of CST1 and increased invasiveness

**Figure 3:**
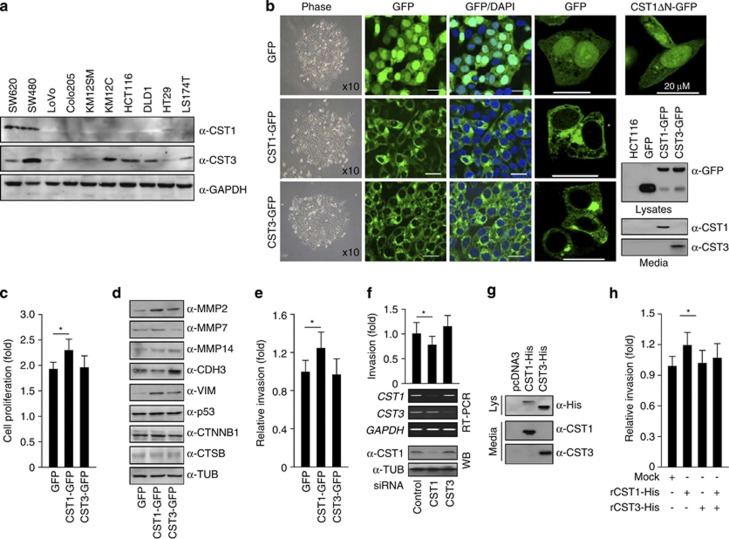
Cellular localization and secretion of CST1 and increased invasiveness of CST-overexpressing cells. (**a**) CST1 protein levels in CRC cell lines. Cells were lysed, and western blotting was conducted. (**b**) Cellular morphology and distribution of CST1. Stable HCT116-CST1-GFP and HCT116-CST3-GFP cell lines were generated using G418 selection and were examined using confocal microscopy and western blotting. (**c**) Increased proliferation of CST1-GFP cells. After 2 days, CST1-GFP and CST3-GFP cells were treated with WST1, and the OD_450_ was read. (**d**) Increased MMP levels in CST1-cells. CST1-GFP and CST3-GFP cells were lysed, and western blotting was performed. (**e**) Increased invasiveness in CST1-cells. Cells were CST1-GFP, and CST3-GFP cells were transferred to the upper wells of a 24-well plate-based invasion assay kit, and after 2 days cells in the lower wells were assayed as described in the Materials and Methods section. The invasiveness of CST1-GFP cells was increased by 20%. (**f**) Decreased invasion in CST1-suppressed cells. SW620 cells were transfected with siRNA against CST1 or CST3 and, after 3 days, were lyszed or transferred to the invasion assay kit as in panel (**e**). (**g**) Purification of CST1-His. Conditioned media from stably generated HCT116-CST1-His and HCT116-CST3-His cells lacking FBS were collected, and secreted CST1-His and CST3-His proteins were purified using Ni-beads. (**h**) The increased invasiveness induced by CST1 was blocked by CST3. HCT116 cells were transferred to the upper wells of an invasion kit and treated with 0.3 *μ*g/ml of recombinant CST1 (rCST1-His) or rCST3-His, and after 2 days cells in the lower wells were assayed. The mean±S.D. from three independent experiments performed in triplicate are shown. *P<0.05

Recombinant native CST1 (rCST1) protein was produced using the mammalian Freestyle 293 Expression System and immunized into BALB/c mice for anti-CST1 antibody production. To examine the protein expression and antibody specificity for CST1, CRC cell lines (Figure 3a) and HEK293-CST1 or HEK293-CST3 stable cell lines (Supplementary Figure S1A) were analyzed by western blotting with anti-CST1 antibody. To analyze the reason for CST1-overexpression in CRC tissues, stable CST1-overexpressing cell lines were generated. As shown in Figure 3a, several CRC cell lines showed little or no expression of CST1 protein. In contrast, CST3 was ubiquitously, albeit differentially, expressed. HCT116 cells were transfected with pEGFPN2-CST1, pEGFPN2-CST3, or vector only, and stable cell lines were selected using G418 treatment. CST1-GFP-, CST3-GFP-, and GFP-expressing HCT-116 cells were analyzed by confocal fluorescence microscopy and western blotting assays (Figure 3b). C-terminally GFP-fused cystatins were highly expressed as full-length proteins, and cleaved-products were also detected (western bloting in Figure 3b). As expected, CST1- and CST3-GFP fusion proteins were detected in the conditioned media. Interestingly, CST3-GFP cells were more adherent to each other, but CST1-GFP cells showed non-adherent growth, much like the HCT116 parent cells (Figure 3b, Phase). CST3-His expression in HEK293 cells resulted in adhesive growth (Supplementary Figures S1B and C), indicating that the more adhesive HEK293-CST3-His cells migrated more slowly and their growth was retarded more than the CST1-His cells (Supplementary Figure S1D). In HCT116 cell lines, cell proliferation increased more in CST1-GFP cells than in CST3-GFP cells (Figure 3c). When we analyzed their cellular distribution using confocal microscopy, CST1 and CST3 appeared to localize to the endoplasmic reticulum–Golgi apparatus (Figure 3b). It is likely that type-2 cystatins containing the secretory leader peptide are transferred to the extracellular space through exocytotic (or secretory) vesicles that originate from the endoplasmic reticulum–Golgi complex. Expression of CST1ΔN33-GFP, in which the N-terminal leader peptide was deleted, showed a dispersed expression pattern, suggesting that secreted CST1 might have a role in the extracellular space.

To examine proteins that were affected by CST1 overexpression, several tumorigenic proteins and mRNAs were analyzed using western blotting and qRT-PCR. As shown in Figure 3d, CST1-GFP cells displayed increased levels of matrix metalloproteinases (MMPs), including MMP2, MMP-7, and MMP-14, as well as vimentin, but CST3-GFP cells revealed a lower MMP expression pattern in comparison. The protein levels of CTSB, p53, and *β*-catenin were not affected. MMP9 and E-cadherin, although weakly expressed (data not shown), were also unaffected. P-cadherin (CDH3) was strongly expressed in CST3-GFP cells, suggesting a relationship to the adhesive growth of CST3-cells. Real-time qRT-PCR demonstrated that the mRNA levels of lysosomal cysteine proteases such as CTSB, CTSH, CTSL, and CTSV were not affected (data not shown). CST1-GFP cells showed a 20% increased invasion activity compared with control cells, whereas CST3-GFP cells were similar to control (Figure 3e). In highly metastatic SW620 cells, a small interfering RNA (siRNA) against *CST1* (siCST1) was used to suppress the expression of *CST1* (Figure 3f). After the expression of *CST1* was detected by RT-PCR and western blotting, cells were subjected to an invasion assay. Suppressing *CST1* resulted in a 20% reduction in invasion activity compared with control cells, whereas suppressing *CST3* produced a slight increase. Alternatively, stable HCT116-CST1-His and HCT116-CST3-His cell lines were generated, as in the GFP cell lines (Figure 3g). Native CST1-His and CST3-His proteins were purified from conditioned media lacking fetal bovine serum (FBS), and their involvement in cellular invasion was examined (Figure 3h). CST1-His cells showed a 17% increased invasiveness, but CST3-His cells were unaffected. Co-transfection with CST1-His and CST3-His resulted in a reduced invasion activity compared with CST1-His cells, indicating that the effect of CST1-His on cellular invasion was blocked.

### Stronger interaction between CST1 and CST3 than between CST3 and CTSB

**Figure 4:**
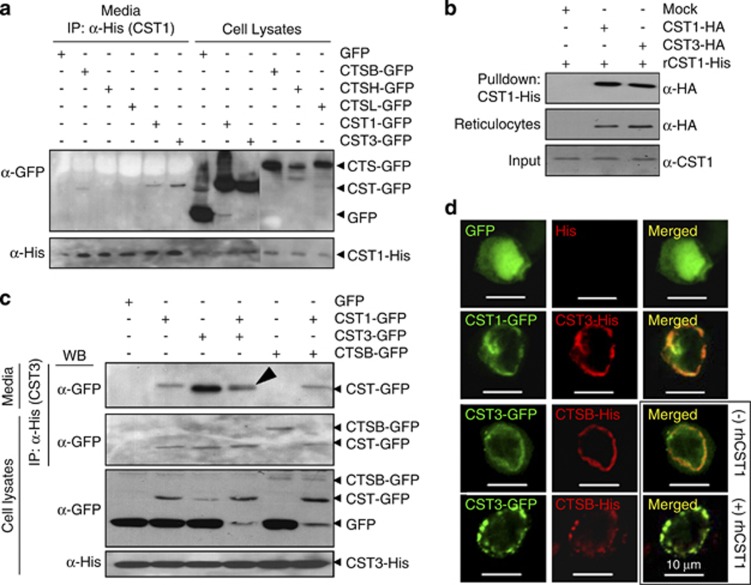
Stronger interaction of CST1 with CST3 than that of CST3 with CTSB. (**a**) CST1–CST3 interaction in the extracellular space. HCT116-CST1-His cells were transfected with pEGFPN2 constructs (CST1-GFP, CST3-GFP, CTSB-GFP, CTSH-GFP, CTSL-GFP) for 2 days. Immunoprecipitation (Sever *et al*^10^) was performed with anti-His antibody in conditioned media followed by western blotting. CST1-GFP and CST3-GFP were precipitated with CST1-His. (**b**) *In vitro* protein interaction of CST1 and CST3. CST1-HA or CST3-HA produced from a reticulocyte system was incubated with immobilized rCST1-His. Precipitates were resuspended and subjected to SDS-PAGE followed by western blotting. (**c**) CST3 interacts with CST3, CST1, and CTSB. After HCT116-CST3-His cells were transfected with pEGFPN2-constructs (CST1-GFP, CST3-GFP, CTSB-GFP), immunoprecipitation of cell lysates and conditioned media was conducted using anti-His antibody. Arrowhead (lane 4) shows two bands (CST1-GFP, CST3-GFP), and binding of CST3-CTSB (lane 5) was not seen in the CST1-expressing cell lysates (lane 6). (**d**) Cellular colocalization of CST1-CST3 and CST3-CTSB. HCT116 cells were cotransfected with pcDNA3.1MycHis and pEGFPN2, and cells were incubated with or without rCST1. Two days later, cells were fixed, stained, and analyzed using a confocal microscope. The fourth row shows that the colocalization of CTSB and CST3 was weakened by addition of rCST1

Type-2 cystatins, including CST1 and CST3, have highly conserved amino-acid sequences at the N-terminus and two β-hairpin loops, L1 and L2,^29, 30^ although the overall identity between CST1 and CST3 is only 50%. We next examined whether CST1 and CST3 interact with each other to form heterodimers. GFP fusion proteins (CST1-GFP, CST3-GFP, CTSB-GFP, CTSH-GFP, CTSL-GFP) were exogenously overexpressed in HCT116-CST1-His cells, and conditioned media were immunoprecipitated with anti-His antibody followed by western blotting. As shown in Figure 4a, secreted CST1-His was bound to CST1-GFP, but was bound more strongly to CST3-GFP, suggesting that CST1 forms homodimers with itself and heterodimers with CST3 in the extracellular space. Interestingly, cleaved CTSB-GFP was weakly detectable in CST1-His immunoprecipitates. To verify the direct heterodimeric interaction between CST1 and CST3, we performed an *in vitro* protein-binding assay using a T7 reticulocyte lysate system. CST1-HA or CST3-HA produced from the reticulocyte system was incubated with purified recombinant CST1-His (rCST1-Hs), which was immobilized on Ni-NTA resin. Figure 4b shows the strong interaction between CST1 and CST3, indicating that CST1 can indeed form a heterodimer with CST3.

Next we examined whether CST1 can compete with the interaction of CST3 and CTSB, as CST3 interacts strongly with CTSB. To analyze their competitive interaction, GFP fusion proteins (CST1-GFP, CST3-GFP, CTSB-GFP) were exogenously overexpressed in HCT116-CST3-His cells, and cell lysates and conditioned media were immunoprecipitated with anti-His antibody (Figure 4c). CST3-His bound to CST1-GFP and also strongly bound to CST3-GFP. Interestingly, when CST3-His was immunoprecipitated with conditioned medium containing CST3-GFP and CST1-GFP, the interaction of CST3-His and CST3-GFP was weakened, as shown by two bands (CST1-GFP and CST3-GFP) in the competitive interaction (Figure 4c, lane 4, indicated by arrowhead). These results suggest that the heterodimeric interaction of CST1 and CST3 is stronger than the homodimeric interaction of CST3. CTSB-GFP interacted with CST3-His in the cell lysates but not in conditioned medium (lane 5). However, association of CST3 and CTSB was not observed in the CST1-containing lysates or conditioned medium, whereas the CST1–CST3 interaction was detected in both cells lysates and conditioned medium (lane 6). These data show that the heterodimeric interaction of CST1 and CST3 is stronger than the binding of CST3 to CTSB, suggesting that CST1 may neutralize the inhibitory effect of CST3 on CTB activity. To verify the interaction between CST1, CST3, and CTSB, confocal microscopy was used to verify their cellular colocalization (Figure 4d). CST1, CST3, and CTSB localized to the extracellular membrane, and colocalization of CST1 and CST3, as well as CST3 and CTSB, was seen (yellow and pink). When rCST1 was added to cells co-expressing CST3-GFP and CTSB-His, the colocalization of CST3 and CTSB was weaker than in cells without rCST1.

### CST1 neutralizes the inhibitory effect of CST3 on CTSB activity

**Figure 5:**
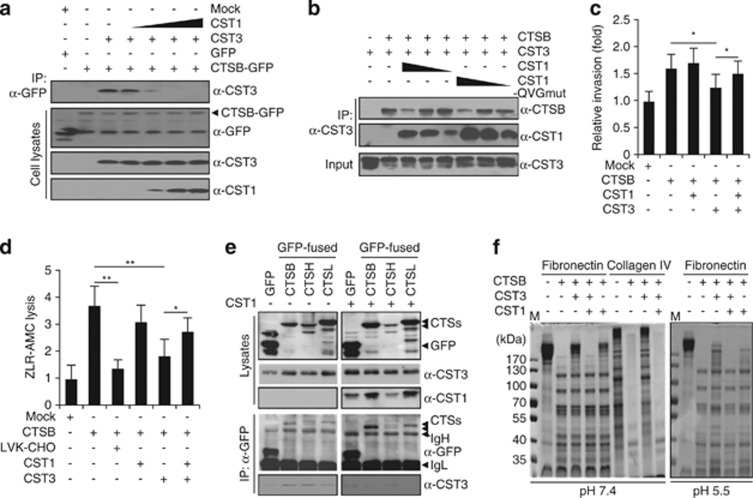
CST1 neutralizes the inhibitory effect of CST3 on CTSB activity. (**a** and **b**) Stronger binding motif of CST1 to CST3 than that of CST3 to CTSB. HCT116 cells were cotransfected with pcDNA3.1-CST1, -CST3, and pEGFPN2-CTSB for 2 days, and cell lysates were immunoprecipitated with anti-GFP antibody. Blocking of CST3–CTSB binding affinity depended on CST1 expression (**a**). HCT116 cells were cotransfected with pcDNA3.1 constructs, and cell lysates were immunoprecipitated with anti-CST3 antibody. CST3–CTSB binding was blocked by wild-type CST1 and the QVG mutant (**b**). (**c** and **d**) CST1 neutralizes the CST3 activity. Cells were co-transfected with pcDNA3.1-CST1, pcDNA3.1-CST3, and pcDNA3.1-CTSB plasmids and, after 1 day, were transferred to the invasion kit. Decreased invasion induced by CST3 was recovered by CST1 (**c**). Cells were transfected as in panel (**c**), and cells with or without LVK-CHO were treated with ZLR-AMC, as described in the Materials and Methods section. Proteolytic activity was measured using a fluorometer. Mean±S.D. values from three independent experiments performed in triplicate are shown. *P<0.05 and **P<0.01. (**e**) HCT116-CST3 cells were cotransfected with pcDNA3.1-CST1, pEGFPN2-CTSB, -CTSH, and -CTSL for 2 days, and cell lysates were immunoprecipitated with anti-GFP antibody. CST1 blocked the binding of CST3-Cathepsins. IgH,immunoglobulin heavy chain; IgL, immunoglobulin light chain. (**f**) Recovery of CTSB activity. CTSB was added to Fibronectin or collagen IV containing CST3 with or without CST1, as described in the Materials and Methods section. The reaction mixtures were subjected to SDS-PAGE followed by Coomassie Brilliant Blue staining. M, marker (kDa)

We next determined the role of CST1 upregulation in colon cancer and the function of the interaction between CST1 and CST3 in the extracellular space. To verify the competitive interaction between CST1, CST3, and CTSB, HCT116 cells were cotransfected with pcDNA3.1- and pEGFPN2 constructs, and their cell lysates were immunoprecipitated. As shown in Figure 5a, the binding affinity between CST3 and CTSB-GFP was strong, but it was decreased in the presence of CST1 expression. Similarly, when cell lysates were immunoprecipitated with anti-CST3 antibody, CTSB was strongly bound to CST3 (Figure 5b). However, the presence of CST1 significantly decreased the binding affinity between CST3 and CTSB. CST1-QVGmut, a mutant of the QxVxG motif in the protease inhibitory active site of cystatins, was also effective for blocking CST3–CTSB binding.

The increased invasive activity induced by CTSB overexpression was inhibited by CST3 (Figure 5c), but co-expression of CST1 and CST3 disrupted this inhibition (*P*<0.05). The proteolytic activity of CTSB in cells was measured using a fluorogenic peptide substrate, Z-Leu-Arg-AMC, which has been used to assay human cathepsins B, L, and V. CTSB proteolytic activity was markedly lower in cells treated with LVK-CHO, a potent CTSB inhibitor (Figure 5d). CST3-overexpressing cells showed a strong reduction in CTSB activity, but this suppression was abrogated by approximately 50% upon CST1 expression.

Also, the relationship between CST3 and the lysosomal cathepsins, including CTSB, CTSH, and CTSL, was examined. As shown in Figures 2b and 4d and Supplementary Figure S2, CST3 was localized at extracelluar matrix and internalized to lysosomes.^31^ Although CST3 and CST1 were partially cololocalized with LAMP1 (lysosomal-associated membrane protein 1), a lysosomal marker protein (Supplementary Figure S2), whether CST3 can inhibit lysosomal cathepsins was examined. HCT116-CST3 cells were cotransfected with pcDNA3.1-CST1 and pEGFPN2-CTSB, -CTSH, and -CTSL, and cell lysates were immunoprecipitated with anti-GFP antibody (Figure 5e). CST3 was bound to CTSB, CTSH, and CTSL; however, CST1 blocked their interaction as shown in Figures 4 and 5. Furthermore, when recombinant fibronectin or collagen IV, extracellular matrix proteins, was incubated with recombinant CTSB, CST3, or CST1, the proteolytic activity of CTSB was significantly blocked by CST3 at pH 5.5–7.4, and its inhibition was disrupted in the presence of CST1 (Figure 5f). This suggests that CST3 inhibits the ECM degradation by the proteolytic activity of CTSB and CST1 neutralizes the inhibitory effect of CST3.

### Conservation of CST1 conformation is important for its secretion and its neutralization of CST3 activity

**Figure 6:**
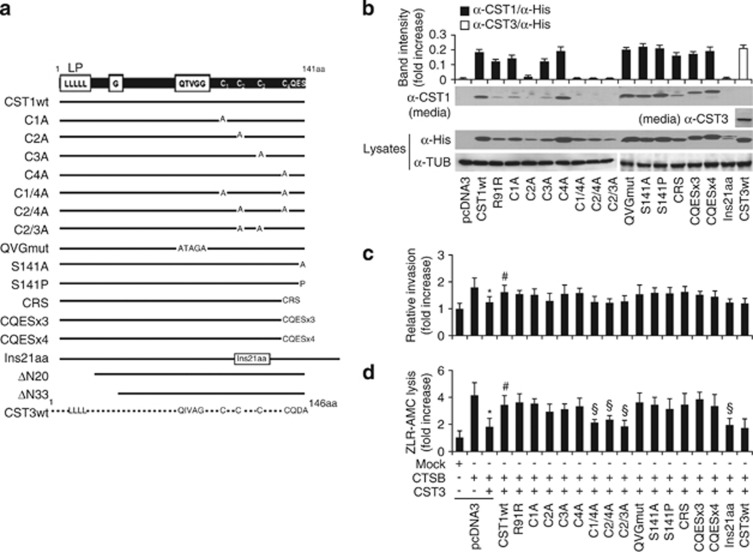
Conserved conformation of CST1 is important for its secretion and its ability to neutralize CST3 activity. (**a**) Scheme of CST1 or CST3 mutagenesis. CST1 (141 amino acids), containing a Leu-rich leader peptide, four Cys residues (C1–4), and a QxVxG motif, was mutated at various sites, as indicated. (**b**) HCT116 cells were transfected with various pcDNA3.1MycHis-CST mutants, and cell lysates (Lys) and conditioned media were examined by western blotting. Band intensity shows the relative density of CST1 in conditioned media compared with cell lysates. (**c** and **d**) CST1 neutralizes the inhibitory effect of CST3 on CTSB activity. HCT116 cells were transfected with pcDNA3.1-CTSB, pcDNA3.1-CST3, and pcDNA3.1-CST1 mutants, and cells were analyzed for invasion (**c**) and proteolysis (**d**). Mean±S.D. values from three independent experiments performed in triplicate are shown. *P<0.05 *versus* CTSB only; ^#^*P*<0.05 *versus* CTSB+CST3; ^§^*P*<0.05 *versus* CTSB+CST3+CST1wt

CST1 has a Leu-rich secretory leader peptide at its N-terminus, a central QxVxG consensus sequence, and four Cys residues that form two disulfide bonds at the C-terminus. To examine which regions of CST1 are important for secretion and protein interaction, CST1 was characterized using various deletions and point mutations. One or two of the four Cys residues were substituted with Ala, the cysteine protease inhibitory QxVxG motif was mutated to AxAxA, or the C-terminal CQES residue was modified to CQEA, CQEP, CRS, or a tri- or tetra-CQES repeats (Figure 6a). In addition, the N-terminal secretory leader peptides were deleted (CST1-ΔN20 and -ΔN33). Before the molecular characterization of CST1, HCT116 cells were transfected with various CST1 mutants, and their expression levels were verified (Figure 6b). We then examined whether these mutants of CST1 affected the ability of CST3 to regulate CTSB. Whole cell lysates and conditioned media showed expression of the CST1 mutant, but their expression levels were different. Although cells were transfected with equal amounts of plasmid, differential or unstable expression of the mutant CST1 was likely due to transcriptional, translational, or structural modification. Mutants of a C-terminal Ser residue, (S141A and S141P), tri- or tetra-CQES repeat mutants, and CRS mutants were detected as wild-type CST1, and phosphorylation of the Ser residue was not detected in our study.

Interestingly, the four Cys residues were important for the extracellular secretion of CST1, indicating that one or more disulfide bond is needed (Figure 6b, mutants C1/4A, C2/4A, C2/3A, and Ins21aa). As shown in Figures 6c and d, invasiveness and the proteolytic activity of CTSB were blocked by CST3 expression, and its inhibition was significantly neutralized by CST1 co-expression (*P*<0.05). Mutants in which two of the four disulfide bond-forming Cys residues were substituted with alanine (C1/4A, C2/4A, C2/3A) and a construct in which an arbitrary 21 amino acids were inserted into the C2 C3 region did not have a neutralizing effect on CST3, suggesting that a disulfide-bonded conformation or conserved folding of CST1 may be important for neutralizing the CST3 activity.

## Discussion

Little is known about the role of CST1 in cancer, but elevated expression of cystatins is associated with tumor invasion and metastasis. In particular, CST3 is involved in tumor invasion and metastasis,^23, 24^ of thyroid carcinomas,^32^ colorectal tumors,^25^ and non-Hodgkin B-cell lymphomas.^33^ CST6 and CST7 are differentially expressed in metastatic mammary epithelial cells^34^ and in metastatic squamous carcinomas.^26, 27^ Polymeric cystatin nanoparticles can effectively inhibit CTSB protease activity,^35^ and the inhibition of cathepsin activity by a pan-cathepsin inhibitor, JPM-OEt, enhances chemotherapy regimens by decreasing tumor growth and invasiveness.^8^ Therefore, highly upregulated cystatins or cathepsins are important for early- and late-stage tumor cell progression, and a balanced regulation between cysteine proteases and cystatins may be clinically useful as an anti-cancer therapy.

CST1 and CST3 have conserved amino-acid sequences and two β-hairpin loops, L1 and L2.^29, 30^ The loop L1 and the C-terminal region of CST1 are important for inhibiting the papain cysteine proteases.^19, 21, 36^ As shown in Figure 6, domain mapping of CST1 revealed that the disulfide-bonded conformation, or conserved folding, of CST1 is important for its secretion and ability to neutralize CST3. The neutralizing effect on CST3 activity was lost in CST1 mutants, including the Cys mutants with non-native conformations, which indicates that the two loops containing four Cys residues are important for CST1 functions. In physiological conditions, CST3 is found as a monomer, but under crystallization conditions CST3 forms domain-swapped dimers.^37, 38^ The L68Q variant of CST3 spontaneously forms dimeric structures via domain swapping, leading to amyloid deposits in the cerebral vasculature, such as those found in hereditary cystatin C amyloid angiopathy or Alzheimer's disease.^39, 40^ In our study of colon and gastric cancers, a CST3 mutation was not found, but a silent mutation at Arg-91 (R91R) in CST1 was identified in both cancers (Figure 1b), although its relevance is not yet understood. In recent report, a silent mutation in a complex membrane transport protein, MDR1, altered its substrate specificity.^41^ CST1 mutation may change the conformational structure motif of substrate and interaction site of inhibitor.

CST3 has long been known as a potent inhibitor of CTSB,^22^ but we showed that the interaction between CST1 and CST3 was stronger than the interaction between CST3 and CTSB (Figures 4 and 5). This finding suggests that CST1 might be a strong regulator of CST3 and that heterodimeric CST1–CST3, as well as monomeric or homodimeric CST1 and CST3, may have a role in the regulation of CTSB protease activity. Exogenously overexpressed CTSB increased cancer cell invasion and substrate proteolysis, effects that were inhibited by CST3 (Figures 5c and d). However, the inhibitory effect of CST3 on CTSB activity was reduced by 50% through the effects of CST1 (Figures 4, 5, 6). Kos *et al.*^42^ reported that the level of CTSB was significantly increased in sera of patients with CRC and a correlation between CTSB serum level and advanced Dukes' stage was found. Also, they showed that CRC patients with high levels of CST3 exhibited a significantly higher risk of death^25^ and that the levels of CTSB and CST3 were significantly higher within the group of metastatic melanoma patients, suggesting that CST3 may be elevated in malignant sera to balance the increased values of CTSB.^43^ It is likely that the balance of CST3 and CTSB affects CRC tumorigenesis, although various cathepsins and cystatins would be regulated depending on the cellular circumstances. We therefore propose that CST1 may contribute to the dissociation of the CST3–CTSB complex by competitive heterodimeric CST1–CST3 binding, and CST1 would have beneficial effect to CRC patients. Although the exact molecular mechanisms of action of cystatins and cysteine proteases in cancer progression are still unclear, a balanced regulation of cysteine proteases by cystatins might be important for tumorigenesis.

This study shows that the upregulation of CST1 in CRC contributes to colorectal tumorigenesis by neutralizing the inhibitory effect of CST3 on CTSB's proteolytic activity.

## Materials and Methods

### Cell culture and plasmids

Human CRC cell lines (HT29, SW480, SW620, Lovo, SNUC1, KM12C, Colo205, SNUC2A, KM12SM, HCT116, LS174T) and gastric cancer cell lines (SNU-1, -16, -216, -620, -638, AGS) were obtained from the Korean Cell Line Bank (Cancer Research Center, Seoul National University, Seoul, Korea). Cells were cultured in RPMI 1640 or Dulbecco's Modified Eagle's Medium (Gibco-BRL, Grand Island, NY, USA), supplemented with heat-inactivated 10% FBS (Gibco-BRL) and antibiotics (100 U/ml penicillin and 100 *μ*g/ml streptomycin), and maintained at 37 °C in an incubator containing a humidified atmosphere of 5% CO_2_. Full-length *CST1* (GenBank acc. no. NM_001898) and *CST3* (NM_000099) cDNAs were isolated from the cDNA of gastric cancer tissue by PCR and cloned into the pEGFPN2/C2 (Clontech, San Francisco, CA, USA) and pcDNA3.1MycHis vectors (Invitrogen, Carlsbad, CA, USA).^28^ Various mutants of *CST1* and *CST3* were generated by restriction enzyme digestion and PCR amplification. Cathepsins B, H, and L cDNAs were isolated from the colon cancer cell line, HCT116, and cloned into pEGFPN2 to create the fusion genes CTSB-GFP, CTSH-GFP, and CTSL-GFP, respectively. All plasmid constructs were verified by automatic DNA sequencing (Bioneer, Daejeon, Korea), and protein expression was determined by western blotting.

### Xenograft assays in nude mice

Six- to 8-week-old male BALB/c athymic nude mice were purchased from Orient Bio. Institute (Seongnam, Korea) and used for the *in vivo* experiments. For all experiments, nude mice were maintained in accordance with the Guidelines and under the approval of the Institutional Review committee for Animal Care and Use (Korea Research Institute of Bioscience and Biotechnology). Two clones of HCT-116 cells stably expressing CST1-GFP and control GFP-transfected cells were used in a xenograft assay. For the xenograft assay, cells were collected by centrifugation, washed twice in phosphate-buffered saline (PBS), 3 × 10^6^ cells were resuspended in 0.1 ml of PBS, and injected s.c. into nude mice (eight mice per cell line). The weights and tumor volumes of the animals were monitored twice weekly. The tumor volumes were measured with calipers and calculated using the following formula: (*A* × *B*^2^)/2, where *A* is the largest and *B* is the smallest diameter.

### RNA extraction and RT-PCR analysis

CRC cell lines and normal/tumor paired tissues from patients with CRC were prepared and lysed using TRI reagent (Molecular Research Center, Cincinnati, OH, USA), and total RNA was isolated according to the manufacturer's instructions. All samples were obtained with informed consent, and their use was approved by the Institutional Review Board of the Eulji University hospital, Daejeon, Korea. After quantification, 5 *μ*g of RNA was annealed to oligo (dT) at 65 °C for 5 min and the RNA-oligo (dT) mixtures were incubated with reverse transcriptase and dNTPs (ProSTAR First-Strand RT-PCR kit; Stratagene, La Jolla, CA, USA) at 42 °C for 1 h. Each cDNA sample was amplified by PCR using Ex Taq polymerase (Takara, Shiga, Japan). Primers used in this study were as follows: *CST1* (sense, 5′-ATGGCCCAGTATCTGAGTAC-3′, antisense, 5′-GGATTCTTGACACCTGGATT-3′); *CST3* (sense, 5′-CCAGCAACGACATGTACCAC-3′, antisense, 5′-ACAGGTGGATTTCGACAAGG-3′); *ACTB* (sense, 5′-GATCATTGCTCCTCCTGAGC-3′, antisense, 5′-ACTCCTGCTTGCGATCCAC-3′); *GAPDH* (sense, 5′-GAGTCAACGGATTTGGTCGT-3′ antisense, 5′- TTGATTTTGGAGGGATCTCG-3′). *ACTB* and *GAPDH* were used as reaction controls. Real-time RT-PCR was performed using a Thermal Cycler Dice Real Time System, and data were analyzed using TP800 software (Takara).

### Transfection and western blotting analysis

For the stable HCT116-CST1 cell line generation, HCT116 cells were transfected with pEGFPN2, pEGFPN2-CST1, pEGFPN2-CST3, pcDNA3MycHis, pcDNA3MycHis-CST1, or pcDNA3MycHis-CST3 using Lipofectamine 2000 reagent (Invitrogen) according to the manufacturer's instructions, and cells were selected by using G418 at a concentration of 600 μg/ml. For western blotting, cells plated at optimal densities were transfected with cDNA plasmids or siRNAs (siCST1: 5′-AUCUAUAACGCAGACCUCAAG-3′, 5′-CUUGAGGUCUGCGUUAUAGAU-3′) for 1–3 days. After washing with PBS, cells were lysed with CelLytic M lysis buffer (Sigma, St Louis, MO, USA) containing a protease inhibitor cocktail (Sigma) on ice for 30 min. Lysates were then cleared by centrifugation, and the protein content in the lysate was quantified via a Bradford assay (Bio-Rad Laboratories, Hercules, CA, USA). Forty micrograms of protein was separated by 10–14% SDS-PAGE and then transferred onto a Hybond polyvinylidene fluoride membrane (Amersham Pharmacia Biotech, Piscataway, NJ, USA). The membranes were blocked with 5% skim milk/PBS for 1 h and incubated with the appropriate primary antibodies and HRP-conjugated secondary antibodies at room temperature. After extensive washing, protein bands were visualized using enhanced chemiluminescence western blotting detection reagents (Amersham).

### Cathepsin activity and invasion assay

Cells in six-well plates (containing 4 × 10^5^ cells/well) were transfected with various plasmids for 1–2 days, and the proteolytic activity of cathepsin was determined using a Z-Leu-Arg-AMC fluorogenic substrate (R&D Systems, Minneapolis, MN, USA), according to the manufacturer's instructions. Cell lysates (70 *μ*g) were prepared, added to a 96-well plate, and incubated with 20 *μ*M of the substrate at 37 °C for 1 h in a 5% CO_2_ incubator. The enzymatic activity was measured with a fluorometer (FilterMax F3, Molecular Devices, Sunnyvale, CA, USA) at 405 nm. Alternatively, for measuring ECM degradation, recombinant CTSB (2 *μ*g, R&D Systems) was added to collagen IV or fibronectin (R&D Systems) containing recombinant CST3 (R&D Systems) with or without recombinant CST1 for 12 h at 37 °C in 50 mM sodium phosphate buffer, pH 5.5 or pH 7.4, containing 1 mM EDTA and 5 mM DTT. The reaction mixtures were boiled and subjected to SDS-PAGE followed by Coomassie Brilliant Blue staining. Invasion assays were performed with a cell invasion assay kit (R&D Systems), according to the manufacturer's instructions. After HCT116 cells were transfected with several plasmids for 1 day, 10^6^ cells/ml in DMEM lacking FBS were placed in the upper wells. Lower wells were filled with DMEM containing 10% FBS. After 48 h, cells on the upper surface of the filter were completely removed, and cells that had passed into the lower wells were incubated with Calcein-AM for 1 h and subsequently measured, at 490 nm using a fluorometer (Molecular Devices).

### Laser scanning confocal microscopy

HCT116 cells were cultured on coverslips in a six-well plate for 1 day and then transfected with pEGFPN2 and/or pcDNA3.1MycHis constructs for 1 day. The cells were then washed with PBS, fixed with 4% paraformaldehyde at room temperature for 20 min, and permeabilized with 0.1% Triton X-100/PBS for 10 min. After washing, cells were blocked with 1% BSA/PBS for 30 min and incubated with polyclonal Lamp1 and monoclonal anti-His antibody for 1 h at room temperature. Finally, cells were incubated with TRITC-conjugated anti-mouse antibody in the dark for 1 h, and the nuclei were stained with DAPI (Sigma). Coverslips containing cells were mounted on glass slides with fluorescent mounting medium (DAKO, Glostrup, Denmark), and the cells were visualized using a laser scanning confocal microscope, LSM510META (Carl Zeiss, Jena, Germany), at × 40 magnification. Confocal images were captured using the Zeiss LSM Image Browser program.

### Statistics

Quantitative data are presented as means±S.D., and statistical significance was assessed by a two-tailed unpaired Student's *t*-test. *P*-values<0.05 were considered significant.

## Abbreviations

GFP: green fluorescent protein
GAPDH: glyceraldehyde-3-phosphate dehydrogenase
JPM-OEt: ethyl (2S,3S)-3-[[(2S)-1-[2-(4-hydroxyphenyl)ethylamino]-3-methyl-1-oxopentan-2-yl]carbamoyl]oxirane-2-carboxylate
DAPI: 4',6-diamidino-2-phenylindole
TRITC: tetramethylrhodamine-5-(and-6)-Isothiocyanate
