# Supplementary figures and images for: Cystatin SN neutralizes the inhibitory effect of cystatin C on cathepsin B activity
Source: Cell Death Dis. 2013 Dec 19;4(12):e974–. doi: 10.1038/cddis.2013.485 (PMC3877556; doi:10.1038/cddis.2013.485)

**A**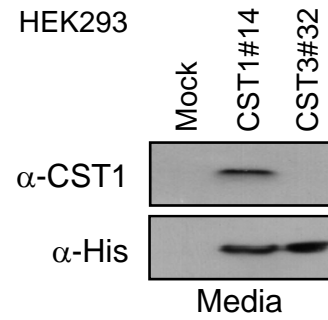**B**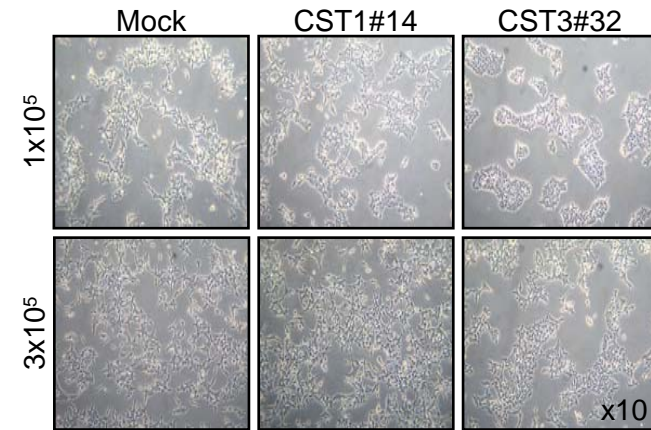**C**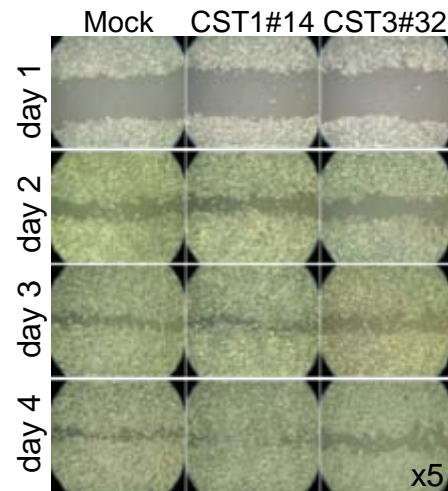**D**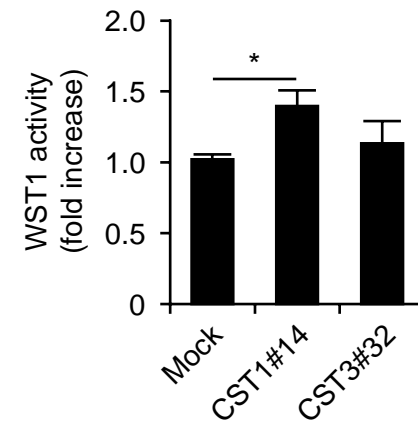

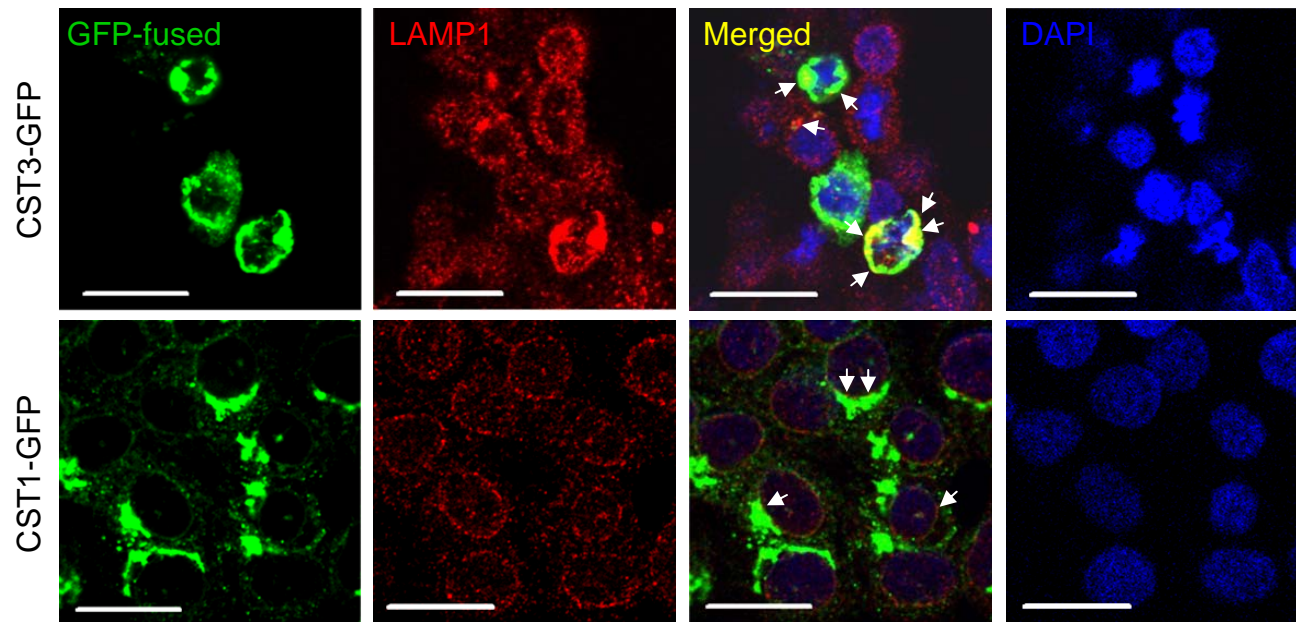

Supplementary Fig 2

Supplement: Supplementary Figures [file cddis2013485x1.pdf]
