# Supplementary material for: Cystatin SN neutralizes the inhibitory effect of cystatin C on cathepsin B activity
Source: Cell Death Dis. 2013 Dec 19;4(12):e974–. doi: 10.1038/cddis.2013.485 (PMC3877556; doi:10.1038/cddis.2013.485)
Supplement: Supplementary Figures Legends [file cddis2013485x2.doc]

**Supplementary Figure legend**

**Fig. S1.** CST1 is involved in cell proliferation, but CST3 contributes to adhesion and retarded cell growth. (A) HEK293-CST1-His cell line generation. HEK293 cells were transfected with pcDNA3.1MycHis-CST1 or pcDNA3.1MycHis-CST3, and cells were selected using G418 treatment. Conditioned medium lacking FBS was collected from each cell line, and Western blotting was conducted. (B) Microscopic images of HEK293-CST1-His and HEK293-CST3-His stable cell lines. Images were obtained 2 days after seeding at 1×10^5^ or 3×10^5^ cells/ml. (C) HEK293-CST1-His and HEK293-CST3-His cells were plated in 100 mm plates, and, 2 days later, monolayers of cells were scratched with a 1 ml pipette tip. Cells were photographed at the indicated days. (D) Increased cell proliferation in HEK293-CST1-His cells. HEK293-CST1-His and HEK293-CST3-His cells in 96-well plates were treated with WST1, and their intensities were measured. Mean ± SD values from three independent experiments performed in triplicate are shown. * *P*<0.05.

Fig. S2. Lysosomal interalization of CST1 and CST3. After transfected with CST1-GFP and CST3-GFP overexpression plasmid, CST1-GFP and CST3-GFP cells were fixed, permeabilized, and stained with LAMP1 antibody for lysosome and DAPI for nucleus. When cells were transiently transfected with CST3-GFP plasmid, the colocalization of CST3-GFP and LAMP1 was increased. All panels; magnification (x400).
